# Are clinical genomes already becoming semi-routine for patient care?

**DOI:** 10.1186/gb-2011-12-s1-i3

**Published:** 2011-09-19

**Authors:** Mark Boguski

**Affiliations:** 1 Harvard Medical School, Boston, MA, USA

## 

Hardly a month goes by without a new published report of a patient’s genome being used diagnostically for clinical management in a diverse spectrum of disease areas, including gastroenterology, nephrology, neurology and oncology. The impression is that clinical genomics is already becoming semi-routine. However, a large and complex set of non-technical barriers needs to be overcome before genomics can truly be integrated into the practice of medicine and made widely available for patient care. Through the use of case studies, my presentation will elucidate issues relating to the needs and requirements of the workforce, the legal and regulatory aspects of ‘laboratory-developed tests’ and insurance reimbursement for ‘multi-analyte diagnostics’. The roles of the Food and Drug Administration, the Centers for Medicare & Medicaid Services and the College of American Pathologists will be highlighted.

